# A Multi-Modal Person Perception Framework for Socially Interactive Mobile Service Robots

**DOI:** 10.3390/s20030722

**Published:** 2020-01-28

**Authors:** Steffen Müller, Tim Wengefeld, Thanh Quang Trinh, Dustin Aganian, Markus Eisenbach, Horst-Michael Gross

**Affiliations:** Neuroinformatics and Cognitive Robotics Lab of Technische Universität Ilmenau, 98684 Ilmenau, Germanyquang-thanh.trinh@tu-ilmenau.de (T.Q.T.); dustin.aganian@tu-ilmenau.de (D.A.); markus.eisenbach@tu-ilmenau.de (M.E.)

**Keywords:** multi modal person tracking, sensor fusion, user centered robot navigation

## Abstract

In order to meet the increasing demands of mobile service robot applications, a dedicated perception module is an essential requirement for the interaction with users in real-world scenarios. In particular, multi sensor fusion and human re-identification are recognized as active research fronts. Through this paper we contribute to the topic and present a modular detection and tracking system that models position and additional properties of persons in the surroundings of a mobile robot. The proposed system introduces a probability-based data association method that besides the position can incorporate face and color-based appearance features in order to realize a re-identification of persons when tracking gets interrupted. The system combines the results of various state-of-the-art image-based detection systems for person recognition, person identification and attribute estimation. This allows a stable estimate of a mobile robot’s user, even in complex, cluttered environments with long-lasting occlusions. In our benchmark, we introduce a new measure for tracking consistency and show the improvements when face and appearance-based re-identification are combined. The tracking system was applied in a real world application with a mobile rehabilitation assistant robot in a public hospital. The estimated states of persons are used for the user-centered navigation behaviors, e.g., guiding or approaching a person, but also for realizing a socially acceptable navigation in public environments.

## 1. Introduction

In recent years, mobile interactive service robots have been developed to operate in private home environments as personal assistants (see [1] for a recent survey on home service robots), and in public places, such as airports [2] and office buildings [3], as receptionists [4] and guides [5]. For such systems, adequate perception skills regarding the persons in the robot’s proximity are essential to fulfill their individual tasks. For some applications, such as those of infotainment robots, it might be enough to detect the presence of a potential user in an image or even identify a face found in a single image. Other applications require a more complex analysis of the scene in the vicinity of the mobile robot in order to adapt the navigation behavior accordingly [6], especially when the robot is operating in a populated public environment.

**Figure 1:**
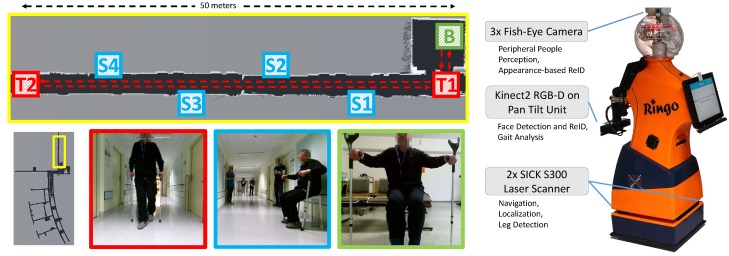
Application scenario in a rehabilitation clinic. Patients are escorted by the robot (on the right) while their gait patterns are analyzed in order to give advice for improving the self-training. Walks were along a populated aisle between T1 and T2, after the session started at B. At any time, the patient can take a rest at the provided chairs at S1–S4; that has to recognized by the robot in order to go to a waiting position until the training is continued.

The work presented here was part of the research project ROGER (RObot-assisted Gait training in orthopEdic Rehabilitiation) [7] in which we developed a rehabilitation robot assisting patients to recover their physiological gait after an orthopedic surgery. After the surgery, the patients were encouraged to perform self-training consisting of walking exercises in an aisle of the hospital. But self-training is only effective if the patients are corrected immediately when they deviate from the physiological gait pattern, or are given positive feedback when they walk without incidents. To this end, the robot has to accompany the patients during their self-training and analyze the gait in real-time. Thus, it has to keep a desired distance between 2.5 and 3.5 m in front of the patient in order to keep the subject in the field of view of a RGB-D camera. While the actual analysis of gait patterns is based on skeleton tracking with the Kinect2 RGB-D camera [8,9], the focus of this paper is on the peripheral perception of persons needed for appropriate robot navigation. The hospital is a crowded public space with lots of bystanders, requiring not only a detection of persons in image space, but a consistent model of the 3D positions and properties of all persons in the robot’s vicinity. The limited field of view of the Kinect2 camera is not sufficient for that purpose. Furthermore, it is pointing opposite to the driving direction. Thus, it is necessary to realize a data fusion for several sensors comprising laser range finders and wide-angle cameras (see Figure 1). For this fusion, the individual characteristics of the detectors have to be considered. Laser range finders allow for exact position measurements, but suffer from a high number of false detections, while image-based person detectors have fewer false detections but more uncertain 3D position information. The proposed system solves this problem by probabilistically modeling the detector’s characteristics.

In our scenario, the ability to identify the interacting patient in order to accompany the correct person is of particular importance. The movements of the patient are used to control the robot’s driving speed during the guided training and are also used for triggering the transition from guiding to approaching the patient, if s/he sits down for a break. Approaching is necessary to close the distance to the patient, allowing for physical interactions with the robot’s touch screen. While the robot stands close, the patient is not visible in the Kinect2 camera, but the robot still has to know the patient’s position for correct navigation. Additionally, for realizing a socially acceptable navigation, all the other persons in the surroundings need to be considered. A correct prediction of the movements and intents of those persons requires the analysis of their body poses (standing or sitting), their movement directions and their body orientations in the environment. All those properties have to be modeled, even if the robot’s sensors are not focusing on the respective persons.

According to the comprehensive overview [10], recent topics for research on multi object tracking are scene understanding in combination with tracking, the information fusion from several sensors and the combination of tracking with other computer vision tasks, such as human re-identification. Our work, in particular, covers the re-identification and sensor fusion topics, and therefore, enables the application of robots in challenging real-world scenarios.

In this paper, we describe the multi-modal detection and tracking framework, which has been developed to serve as a basis for the social navigation of mobile service robots. Since the ROGER project is not the only application for our robots, the framework has been designed to be modular and easily extendable; i.e., new detectors for persons and their properties can be plugged in, and additional attribute trackers can be included to complete the probabilistic model of the states of persons and automatically improve data association and re-identification.

The contributions of this paper are

- The introduction of a modular multi-modal tracking framework, which realizes the fusion of independent asynchronous detections in different sensors to form a probabilistic model of all persons in the robot’s surroundings.
- The usage of various properties of tracked persons (face and appearance-based features) for an implicit re-identification of persons after tracking interruption. Therefore, a probabilistic data association step is introduced, which is coupling the individual trackers to their independent properties.
- A benchmark on a published multi-modal dataset shows the improvement of tracking consistency when individual features are added to the standard position tracker.

## 2. Related Work

In the computer vision and robotics community, tracking multiple persons within single or multi-sensor setups has undergone extensive research over the last several decades. Following the categorization of the survey presented in [10], tracking approaches can be divided into *online* approaches, i.e., only using sensor data from the past for the estimation, and *offline* approaches, which process a batch of sensor readings. The targeted applications come with different constraints. Computer vision scenarios, such as surveillance, typically make use of offline approaches, because they are less real-time constrained and allow the inclusion of delayed results to better handle ambiguities. Thus, for these scenarios, the actual movement trajectory is of additional importance to find past positions of persons. However, applications involving human–robot interaction (HRI) are tied to immediate results, since dynamic and rapidly changing environments affect the navigation and HRI behavior of the robot, and therefore, the current state of a person is more relevant than past aspects of his/her movement trajectory, which can be better handled by online methods. Linder et al. [11] showed that individual tracking solutions cannot be used out of the box for each application. More elaborated offline tracking approaches like [12] perform on par with simple online filters [13] when parameterized to deliver real-time results. Therefore, in our application field, online filter approaches are preferred for tracking. For tracking time-variant states, optimal Bayes filters dominate the literature [14]. Especially, the Kalman filter and its derivatives Extended Kalman filter (EKF) and Unscented Kalman filter (UKF) can be found in almost every tracking approach for robotic applications. Hence, our implementation makes use of the Bayes filter concept as well.

### 2.1. Sensor Fusion in Mobile Robot Person Tracking

For robotic applications it is often necessary to combine asynchronous data from different cameras and sensors in order to realize a suitable perception range. Therefore, in the following, we concentrate on tracking approaches which have already been deployed in dynamic robotic scenarios with a multiple sensor setup. Approaches for tracking persons from multiple sensor inputs mainly originate from the robotics community. In [15], different filter approaches (EKF, UKF, and Particle Filter) were compared using estimations from a leg and a face detector. Leg detections in laser data and a body depth template detector were used in [13] in conjunction with the same tracking back-end as [15]. Volkhardt et al. [16] tested different combinations of visual face, upper- and full-body detectors in combination with a leg detector as input for a Kalman filter.

All of these approaches have in common that they fuse fast but unreliable laser-based detections having a large range of view with vision-based detectors having better selectivity. In order to combine these data, setups can be organized hierarchically or in parallel each of them having individual pros and cons. In hierarchical systems, faster weak detectors are used to restrict the more elaborate but computationally expensive stronger detectors to promising regions in the input. Unfortunately, missing detections in the first stage of such an approach cannot be corrected in the following levels of the hierarchy. Furthermore, the possibility of increasing the range by using different sensor systems is hard to implement by hierarchical approaches. Therefore, in our approach we have focused on a parallel configuration - detections of all sensors are treated equitable while the individual characteristics of detectors regarding false detection rate and miss rate have to be taken into account.

### 2.2. Multi Target Tracking

Furthermore, the problem of data association, which comes along with multi-target tracking, needs to be solved before the update step of the Bayes filter can be applied. While following a tracking-by-detection approach, for each time step the set of independent detections needs to be assigned to one of the hypotheses in the tracker’s belief, or a new hypothesis needs to be spawned. Approaches for solving the data association problem reach from most straightforward nearest neighbor association, as used in [17], over more accurate methods for maximizing the overall association scores with the Hungarian algorithm, to probabilistic models, like the joint probabilistic data association (JPDA) [18]. The latter uses a soft assignment of detections to tracks. Besides the decision of a single mapping solution, there are also approaches following multiple hypotheses for the associations leading to multi-hypotheses tracking [19]. Unfortunately, the computational effort increases when multiple options are kept providing only a limited gain in accuracy. Another way to improve data association is to take into account additional image features in a probabilistic manner [20]. These additional information on the one hand can be used for matching the belonging of regions of interest in consecutive images, where deep-learning-based classifiers can be trained to identify image pairs of identical or different persons. Alternatively, for each hypothesis an appearance model of the tracked person can be built inside the tracker in order to decide which track fits best to the new detection. This alternative approach is used in our implementation.

### 2.3. Out of Sequence Measurements in Online Tracking

In filtering approaches, variable latencies of the detector modules lead to the problem of out-of-sequence measurements (OOSM). It can occur that the filter already does a belief update with an observation from time $t_{1}$ when a delayed observation arrives from a sensor reading at time $t_{2}<t_{1}$. In this case, causality for the filter is broken, since the Δt is negative. For single target Kalman filter or particle filter trackers, there are various approaches that use a backward prediction of the latest belief in order to do the update correctly with the delayed observations [21]. Nevertheless, the subsequent integration of delayed measurements is not possible in then case of multi-target tracking, when the delayed update may change the data association of measurements that have already been integrated in the estimation. An easy solution to overcome this problem is the recomputation of all updates after a delayed observation, which has only the drawbacks that observations have to be buffered and computational effort increases because states are computed multiple times.

## 3. System Overview

**Figure 2:**
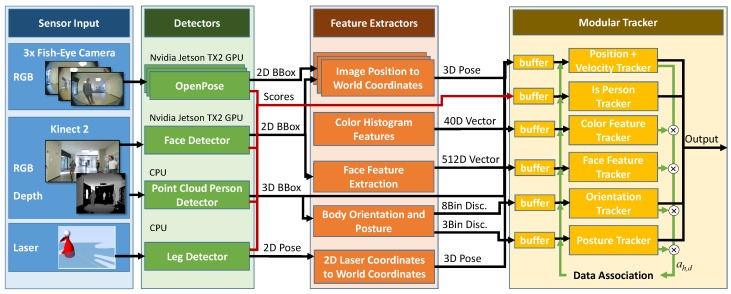
Overview of the modular person detection and tracking system.

Our tracking system can be categorized as a tracking-by-detection approach. On the images of three fisheye cameras, covering a 360° view around the robot, along with the RGB-D data of a Kinect2 and the SICK laser range finder data (see Figure 1), we apply individual detector modules, as shown in Figure 2. The detector modules operate in the domains of their respective input data (image space, point cloud, or 2D plane) producing person detections with a certainty that is expressed in a score. The detection score is transformed into a probability that a detection represents an actual person (called IsPerson probability in the following) by analyzing the resulting detection rates over the scores on a representative test dataset [22]. This probability is used for an adequate consideration of the individual detectors’ false detections and miss rates. Based on the raw detections in the images and point cloud, a set of feature extractors is applied in a second stage. From the detected image regions of presumed persons, these algorithms extract probability distributions of attributes to be tracked in the tracker modules. Finally, the asynchronous streams of these feature detections are sent into the modular tracker, where they are buffered and sorted by the timestamp of the original sensor readings. The tracker itself is responsible for modeling the current state of the persons based on the observations of the past. To keep the system extendable, the tracker is organized in independent modules which can be exchanged without interfering with other modules. In other applications, different attributes might be important, and thus, can be plugged in easily. The data association is done in the core of the tracker, making it possible to use additional information from the other attributes for identification of the hypothesis that belongs. In the following sections, our system will be explained in more detail.

### 3.1. Detection Modules

The most significant detector in our setup is OpenPose [23], a 2D skeleton detector operating on the images of the three fisheye cameras. According to [24], OpenPose outperformed several image-based detectors with respect to detection quality. OpenPose is a CNN-based approach and is only real-time capable if running on a GPU. Thus, on our robot we operate three Nvidia Jetson TX2 GPUs. Each of them is dedicated to one of the wide-angle RGB cameras. On a Jetson TX2, images with a size of 640 × 480 pixels can be processed at 5 Hz, yielding 2D bounding boxes and the 2D position of the body parts of persons (joints) in the image. To enable this run-time, the internal resolution of the network has been scaled down to 336 × 192. Due to the wide opening angle and the low resolution of the fisheye images, the range of this detector is limited to about 5m. In order to cover distances of up to 10m and for generating point cloud segments for further analysis of body orientation and posture analysis, the clustering method from [24] is used on the point cloud extracted from the Kinect2 sensor data.

**Figure 3:**
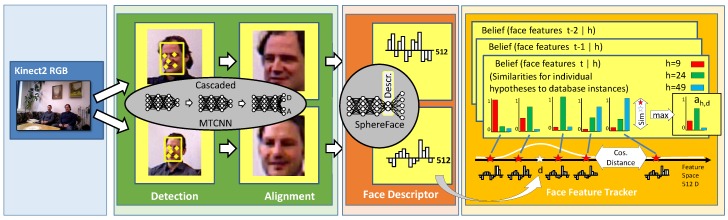
Overview of face-based person detection and identification. Face descriptors extracted for each detected face in the image are used for finding the association probability of detection *d* to the hypotheses *h* in the tracker. Therefore, stored similarities (red, green, and blue bars) of hypotheses to the samples in the feature data base (red stars) get weighted by the cosine similarity of the detected descriptor (white star) to all the sample descriptors. The maximum of the weighted values is used as association probability in the tracker. New observations are used for updating the database and the belief of the new time step as well.

Furthermore, as a prerequisite for the face descriptor extraction step used for re-identification, all faces in the RGB image of the Kinect2 are detected and aligned to a standard geometry (see Figure 3). For face detection and alignment, we utilize a multitask cascaded convolutional neural network (MTCNN) [25], also processed on a Jetson TX2, which consists of the following three networks: The first convolutional neural network (CNN) operates on an image pyramid and is kept simple in order to be fast. It is only four layers deep and has 6632 weights. As output, it predicts proposal regions that may contain faces. The proposal regions are clipped, scaled to 24 × 24 pixels, and processed by the second CNN, consisting of five layers with 100,178 weights. The objective of the second network is to reject some of the non-face regions. The remaining regions are processed with a resolution of 48 × 48 pixels by a third CNN which computes a face recognition score and five landmarks at the eyes, the nose and the corners of the mouth. This network is a bit more complex. It is six layers deep and has 389,040 weights. Using the five landmarks, each face is aligned using a similarity transformation for further processing in the facial feature extraction.

An additional detector processes the range scan data of two horizontal SICK laser range finders, recognizing the legs of persons [26]. The leg detector, with a processing rate of 10 Hz, is relatively fast and yields accurate distance measurements compared to the image-based detectors. The higher update rate is useful for tracking the positions of persons in between image detections, but the reliability of distance scans is significantly weaker compared to the image and point cloud detectors, which causes a number of false detections that have to be handled correctly by the tracker afterwards.

### 3.2. Feature Extraction

According to the processing pipeline shown in Figure 2, the raw detections in all the sensor data are used for extracting the actual properties of interest for the person tracker.

#### 3.2.1. Position in 3D World Coordinates

For the navigation algorithm of our application, the positions of persons represented in 3D world coordinates are important to avoid collisions and interference with their movement trajectories. Therefore, all pose observations going into the tracker are described as 3D Gaussian distributions in world coordinates, which allows for encoding the spatial uncertainty in the covariance matrix. Since detections from the OpenPose and the face detector are only in 2D image coordinates, the distance to the camera needs to be estimated in order to find a 3D position.

We use two methods for determining the distance of the person to the camera. For faces, by assuming that humans have about the same face size, we can derive the distance from the bounding box size of the detected faces in an image. The projective mapping in a camera makes the image size reciprocally proportional to the distance of an object, or in our case the face, of known size. For full body detections, this size-based approach is not applicable due to the large variance of people’s appearances in images depending on posture and occlusions. Instead, similar to [11], a ray is cast through the pixels of the feet (OpenPose yields pixel positions of individual body joints) to the ground plane, yielding a position in world coordinates. Fortunately, persons standing near the robot constitute no problem for our sensor setup, since in the wide-view angle of the used fisheye cameras, the feet of pedestrians are still visible, even at a close distance. By evaluating the position of slightly shifted image coordinates of the human feet, a proper spatial uncertainty can be determined, which grows with the distance to the camera.

The 2D leg detections taken from the SICK laser range scans are already in the xy plane in world coordinates and only need to get assigned an elevation (*z* value). This is the normal distribution of human head height. The 3D bounding boxes of the point cloud detector are interpreted in the same way. They also define a position in xy plane, but the exact height of the head with respect to the box height can only be estimated as an average position representing the prior distribution.

#### 3.2.2. Posture and Orientation

Besides the position, the orientations of persons to be tracked and their postures (standing, sitting, squatting) are important information for making decisions during HRI. Therefore, the detected point cloud segments are further processed by a CNN to classify posture [24] and upper body orientation [27]. The observations of these properties are modeled as discrete distributions with three bins for the posture classes and eight bins for the orientation. Since the used CNN is computationally efficient, it is implemented on the CPU.

#### 3.2.3. Re-Identification

All of these detections and person properties are sufficient for a basic tracking system that does not distinguish persons. This might be sufficient for applications with short-term interactions. In these scenarios, the interaction partner can, for example, be selected based on the proximity to the robot. For long-term interactions, as realized in our training application, the correct users must be recognized even if they disappear for a short moment due to occlusions either by objects or other persons crossing their way. Thus, we extract additional features for re-identification from raw detections in images. The first set of features describes the appearance of each person. Following our proposed approach in [28], we extract color histograms in various color spaces for the full body enclosed by the bounding box in the RGB image. Then, we apply a learned metric to transform the extracted features to a 40 dimensional subspace to allow for fast matching. To compute a suitable feature transformation for distinguishing persons by their appearance under varying environmental conditions, we applied a metric learning approach, namely, local Fisher discriminant analysis (LFDA), on feature vectors transferred to a kernel space using a $\chi^{2}$-RBF kernel [28]. This results in a 40 dimensional feature vector for appearance-based re-identification. At great distance, appearance-based features outperform biometric features due to a low resolution of the person-containing sub-image. Therefore, primarily, we use appearance-based features to identify persons at great distances. At short distance, appearance-based features are applicable too, but since the clothing of persons has limited discriminatory power, biometric features may perform better.

Therefore, at short distance, we use the more distinctive facial features. They are extracted from the full-resolution HD color image of the Kinect2. For each face detected, we extract a feature descriptor by applying a deep neural network following the SphereFace approach [29]. Figure 3 shows that step in the center. The basic idea of SphereFace is to train a network for descriptor extraction by choosing a network topology with a bottleneck, which later becomes the descriptor, and formulate the training as classification with additional restrictions. The network architecture is ResNet-like with 20 convolutional layers followed by the bottleneck of 512 neurons and a softmax classification layer with one neuron for each person in the training set. After training, the classification layer is dispensable, and thus, is dropped. This initial training step is performed offline on the large CASIA-WebFace dataset [30]. It contains 494,414 face images of 10,575 different persons. Due to the information bottleneck in the trained network, the feature extraction generalizes well to persons that have not been observed during training. The resulting generic face descriptor is a 512-dimensional vector that can be compared to others by means of the cosine similarity. The descriptors are trained to some degree, being invariant against changing head orientation and environmental conditions, but trials in our application environment showed that representing a person by a multi-modal model is necessary if illumination conditions change drastically along the movement path of the person. Section 5.3 describes that solution in more detail.

## 4. Multimodal Tracking Framework

The basic idea of our tracking framework is a multi-tracker approach. Each modality—face, pose, position, etc.—is tracked by an individual multi-hypotheses tracker, each sharing a global set of Hypothesis IDs (HIDs). This allows one to combine the belief state of all the trackers for a joint decision on the data association problem. Details on the internal processing and the handling of asynchronous detections are described in the following.

**Figure 4:**
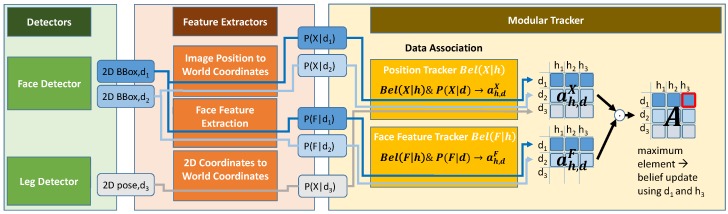
Example for the data flow in the system. The face detector finds two faces and assigns Detection IDs $d_{1}$ and $d_{2}$. In the range scan, one person has been detected ($d_{3}$). The Detection ID is used for assignment of the data association results of the individual tracker modules. Since each module uses a common set of Hypothesis IDs ($h_{i}$), the matrices $A^{X}$ for the position-based association probabilities and $A^{F}$ for the face feature-based association can be multiplied element-wise in order to find the best combination of detection and hypothesis to be used for a belief update.

For decision making in the application and for realizing a highly responsive navigation, it is necessary to consider position information regarding persons as soon as possible. In this context, the 200 ms delay of the image-based detectors and the even longer duration of the face detection and feature extraction pipeline are an issue. The tracking system is designed to run at a fixed cycle time, which was set to 100 ms in our application. Thus, the position and state hypotheses are sent to the navigation system at 10 Hz, which is the internal planning interval of the navigator. In order to always consider the available data at the distinct evaluation times but not skip delayed observations in later state estimations, the tracker modules each have a buffer at the input containing the detections of the last 500 ms (see Figure 2). This buffer size only depends on the latency of the slowest detector. Each buffer is a sorted list keeping the detections in the order of their timestamps, which is usually the original sensor data acquisition time. Therefore, detections with higher latency will be evaluated in the correct order even if faster detections exist. At evaluation time, the belief is rewound to the oldest sensor data timestamp that has arrived in the last cycle interval, and all the detection updates are recomputed up to the current time, as described in Algorithm 1.
**Algorithm 1:** Tracking cycle1**for** all tracking modules **do**2reset belief to begin of rewind interval;3**for** all detection timestamps t in rewind interval **do**4**for** all tracking modules **do**5predict belief using Δt from previous detection;6**while** unprocessed detections at time t **do**7compute matrix element A of all association probabilities to unprocessed detections at t;8find maximum element $a_{h,d}\in A$;9update hypothesis *h* using detection *d*; **end**10**for** all tracking modules **do**11predict belief to current time;12**return** belief state of current time;

The key element of our implementation is a shared set of unique *Hypothesis IDs (HID)* used in each of the individual tracker modules. This allows one to calculate a joint association probability $a_{h,d}$ of a new detection *d* and hypotheses *h* in the tracker (see Figure 4). This binary probability is computed in the individual tracker modules based on their current belief for the respective HID *h* which is compared to the respective detection distribution. In order to be able to combine information of different feature extractors and detectors, the raw detections and features extracted from them get assigned a unique identifier called *Detection ID (DID)* *d*. Together with the commonly used HIDs *h*, this allows one to multiply the association probabilities concerning a specific *d* and *h* from different tracker modules. For example, the association of a face detection in an image can have two supporting tracker modules, the position and the face features voting either for or against a match. There are also detections that do not have additional support from other modules, such as the leg detections. For those, no additional features are computed and tracked; thus, an association is only based on position. The binary association probabilities $a_{h,d}^{m}$ of all modules *m* get multiplied in Bayesian manner:

(1) $$a_{h,d}=\frac{\prod_{m}a_{h,d}^{m}}{\prod_{m}a_{h,d}^{m}+\prod_{m}\left(1-a_{h,d}^{m}\right)}.$$

Equation (1) directly follows as a generalization from the element-wise product of two independent, binary probability distributions and the following normalization. If a tracker module *m* does not have any belief for a certain *h*, $a_{h,d}^{m}=0.5$ represents that no information on the decision is provided. In order to allow for an introduction of new hypotheses in the trackers, each DID *d* is also used as a potential new HID $h_{new}=d$ with an a priori association probability of $a_{h_{new},d}=0.5$. If during the association process no other combination is found that has an association probability greater 0.5, the following belief update is done with that new $h_{new}$, causing the creation of a new hypothesis in the tracker modules.

The main concept for all the tracking modules is a multi-hypotheses Bayes filter estimating the belief state of arbitrary property *m* over time *t* for each HID *h* in the form of a probability distribution $bel\left(m_{t}|h\right)=p\left(m_{t}|h\right)$. Possible properties are either real valued states, such as the position of a person, or categorical states, such as whether a person is sitting. In Section 5, the representation of selected properties is explained in detail. The modules can decide on their own when to delete a hypothesis from their belief, and thus, they implement individual amounts of persistence, reflecting the stability of the modeled attribute. For example, it makes no sense to keep a belief of the person’s position if there are no observations on that property for more than a minute, but depending on the application, color appearance features and also the facial features can be stored for minutes or days respectively. The Bayes filter approach allows one to consider uncertainties in the detections correctly and consists of two main steps. First, to obtain the belief for the current time step, a process or motion model $p(m_{t}|m_{t-\Delta t},\Delta t)$ is applied to the former belief. The prediction model can depend on additional variables (or belief states of other modules; e.g., the posture belief may influence the a priori movement velocity in the position tracker). Second, the predicted belief is updated, which is mainly a multiplication by the probability of the observation $p(o|m_{t})$. For a multi-hypotheses tracker, the data association problem has to be solved before the update, as described above.

### Modeling of Detectors’ Uncertainties

In the proposed tracking system, we want to combine various detection modules, each having different characteristics with respect to detection rates and false alarms. The knowledge of these meta data is often used for the track initialization logic in other systems. Some approaches [13,15] use leaky counters for the number of detections that support a hypothesis or just insert new tracks if a certain motion profile is observed [31]. Other approaches [11,16] insert new tracks immediately, but only consider them as certain if they are confirmed by more than one detector. In [11], different tracking approaches have been compared, and one conclusion was that in a highly dynamic environment there is no optimal solution to handle these problems for every field of application. A liberal strategy to insert new hypotheses could lead to freezing the robot’s social navigation behavior, while an overly conservative strategy would undermine the social acceptance due to misses.

Therefore, we decoupled track initialization from the actual certainty scores of the detections and explicitly modeled a cumulative probability for a HID *h* to represent an actual person. In Figure 2, this is shown in the red pathway. Details of that approach can be found in [32]. The basic idea is to transform the scores generated by the detectors into a real probability of being a true positive detection, which can be done using a ground truth test data set and by counting the ratio of true positive detections for the individual scores. Afterwards, in the tracker module for each detector and each hypothesis, the belief is modeled over time as a binary distribution. A motion model is used, which shifts this probability towards 0.5 with a sensor-specific time constant. This speed of fading out reflects the update frequency of the detector. The probabilities of seeing a real person for all the detectors are finally combined as independent observations by means of the Bayesian product operation, similarly to Equation (1), yielding a value that can be compared with a threshold in the application in order to consider only certain hypotheses. The value of the threshold has been optimized to reflect the point of maximum MOTA (see Section 6).

## 5. Belief Representation in the Individual Tacker Modules

Depending on the natures of the attributes to be tracked and the respective formats of observations generated by the feature extraction modules, the belief representation in the tracker differs. We have implemented multi-variate Gaussian distributions for real-valued properties, discrete distributions for categorical attributes and a special representation for a distribution on high dimensional feature vectors as used for face and appearance-based re-identification. While the first two options follow the standard operations of the Bayes filter [14], the third has been developed to overcome the problems arising with high dimensional spaces. In the following, we briefly want to describe the realization of the individual tracker modules with their internal belief representations and the required operations (belief prediction, computation of data association probabilities and belief update).

### 5.1. Position and Velocity

The position tracker for each HID holds one multivariate Gaussian distribution on the 3D position of a person in world coordinates. Additionally, a 2D Gaussian for the velocity vector in the horizontal plane is stored and both basically updated according to the basic Kalman filter equations [14]. We only limited the velocity and the amount of system noise depending on the belief of the posture tracker. For squatting and sitting persons, the limits are lower compared to standing persons.

The most interesting aspect of the position tracker is the computation of association probabilities. In other multi-target tracking systems, the data association is usually done based on euclidean or Mahalanobis distance of the new detection and the existing hypotheses [33]. In our approach, we adapted this in order to realize the following requirements: First, the spatially closest hypothesis should have the highest probability to be assigned to a new detection, but we wanted to define a distance $r_{max}$ from which it is unlikely that the hypothesis belongs to the detection ($a_{h,d}\leq 0.5$). Second, the shape of the spatial uncertainty of the detection should be considered similar to the Mahalanobis distance for Gaussians. Finally, we searched for a mechanism that prefers new hypotheses over older ones, which can be seen in the variances of the hypotheses. Hypotheses that have been unsupported for a longer time have a larger variance due to the motion model prediction. By associating new observations to more certain hypotheses, the consistency of the tracks can be increased. To that end, we designed a specific, distance-based similarity function $sim(\mu_{h},\Sigma_{h},\mu_{d},\Sigma_{d})$ that uses only the x,y coordinates of the hypotheses’ and the detections’ Gaussians with their means μ and covariances Σ.

(2) $$sim\left(\mu_{h},\Sigma_{h},\mu_{d},\Sigma_{d}\right)=e^{\left(-0.5{\left(\mu_{h}-\mu_{d}\right)}^{T}\Sigma_{d}\left(\mu_{h}-\mu_{d}\right)\frac{|\Sigma_{h}{|}^{\nu }}{r_{max}}\right)}$$

The parameter ν in that equation is used to scale the punishing influence of more uncertainty in hypotheses (by means of the determinant of their covariance).

### 5.2. Posture and Orientation

The orientation and posture of a person are modeled each as a discrete probability distribution in form of a $L_{1}$ normalized vector *b* of probabilities. For the posture, this is due to the categorical character of that property representing standing upright, sitting or squatting. The motivation for modeling the orientation by discrete bins as well (eight in our case) is the possibility for representing multi-modal distributions, which is not possible using parametric distributions of the exponential class. A further problem is the periodicity of a real-valued orientation angle that has to be handled explicitly when the angle is not modeled as a categorical domain. Alternatives for representing the periodical angle in real-valued domain, e.g., as a two dimensional vector, are discussed in [32].

Using the discrete distribution as belief makes the operations of the Bayes filer straightforward. The motion or process model for a discrete distribution is a matrix M of transition rates describing the probability of changing from one state to another in the reference interval of one second. For a given Δt the predicted belief $\hat{b}$ follows from Equation (3) by means of the matrix exponential.

(3) $$\hat{b}=b^{T}e^{\left(\Delta tM\right)}$$

The next operation needed is the calculation of association probabilities. Here, the integral of the product of a hypothesis and a detection distribution can be used, which, basically, is the sum of the element-wise product of the two vectors. This product has a high value if the two distributions are similar. Other options seen in literature are the Kullback–Leibler divergence and the earth mover’s distance. We again had some additional requirements for the similarity. Thus, we designed an additional normalization using the number of bins. The aim of the normalization is to make the resulting association probability equal to 0.5, if one of the operands is a uniform distribution. In such a case, no information on the correctness of the association can be given by this module. Therefore, the association probability for a detection *d* and an hypothesis *h* is computed as:

(4) $$a_{h,d}=n+\left(1-n\right)\sum_{i}b_{i}^{h}b_{i}^{d}.$$

The scaling parameter *n* is $n=\frac{0.5d-1}{d-1}$ with *d* being the number of bins in the distribution.

The operation for the belief update of a discrete belief is a simple element-wise product of the belief distribution and the new observation distribution followed by an $L_{1}$ normalization.

### 5.3. Face and Color Features

In our system, both color and facial feature descriptors are high dimensional vectors *v* (40 D for color and 512 D for face). Modeling a multi-modal probability distribution on these high dimensional feature vectors is not easy. For our system, we developed a sample-based representation which avoids manipulations such as averaging on the actual feature vectors during belief update. The observed feature vectors of the past are used unchanged as a database for identifying new observations using similarity functions known from the person identification domain. Therefore, from all the detections a set of samples $S=\{s_{i},1<i<500\}$ is incrementally built, as shown in Figure 3 in form of the red stars. The overall number of samples is limited by randomly pruning out one of the two most similar samples, if the maximum number (500 in our case) is exceeded. The size of the sample set is only limited by the computation time needed for comparing new observations to all the existing samples. By means of pruning, the sample density in the feature space, in the long run, evens out. Additionally, samples are deleted if they have no assigned hypotheses anymore.

Similarities of two face descriptors can be evaluated based on the cosine distance metric, which is actually the dot product of the normalized descriptor vectors ($d\left(v_{a},v_{b}\right)=1-v_{a}\;\cdot \;v_{b}/|v_{a}\left|\;\right|v_{b}|$).

For the 40 dimensional color feature vectors, the applied metric learning method (see Section 3.2.3) optimizes for a euclidean distances measure, in case the distance is $d\left(v_{a},v_{b}\right)=\left|v_{a}-v_{b}\right|$. In order to transform the distances into probabilities of representing the same individual (similarity), we used data of a test dataset in order to tune the parameters *m* and *c* of a Fermi-function, which pretty much fit the resulting distribution [28,34]. The similarity, therefore, is:

(5) $$sim\left(v_{a},v_{b}\right)={\left(1+e^{\left(\frac{d(v_{a},v_{b})-m}{c}\right)}\right)}^{-1}.$$

In order to represent a probability distribution of descriptors for individual HIDs *h* in the time variant belief, the observed similarities are stored for the existing samples ($s_{i}\times h\mapsto sim\left(s_{i},v_{h}\right)=k_{i,h}$). These are the colored bars in the belief boxes in Figure 3. Using this representation, the operations needed are implemented as follows:

Since the properties of face and color appearance are not time-variant, a motion update is not necessary in the prediction step and is simply left out.

The determination of an association probability $a_{h,d}$ for a detection *d* to a hypothesis *h* is done by comparing the feature descriptor $v_{d}$ of the detection to all the samples $s_{i}$ in our data base by means of the similarity $sim(v_{d},s_{i})$ (Equation (5)). The desired $a_{h,d}$ is the maximum of the stored associations weighted by the similarity.

(6) $$a_{h,d}=\max_{i}\;k_{i,h}\;sim\left(v_{d},s_{i}\right)$$

The update of the belief, given a new observation $v_{d}$ and a belonging HID *h*, is done based also on the similarity of the $v_{d}$ to the samples in the data base. First, the new descriptor is added to the sample set *S* if its distance to every existing $s_{i}$ exceeds a threshold. Then the pruning is done, if necessary. Afterwards, the $k_{i,h}^{t}$ in the belief are to be updated in order to slowly converge towards the observed similarity. Values cannot be set directly to the observed similarities, in order to avoid teaching individual false detections in one shot. The new $k_{i,h}^{t}$, thus, are set to the maximum of the old value and the actual similarity of the detection to the sample scaled with a free parameter τ, which determines the update speed in the model.

(7) $$k_{i,h}^{t}=\max\left\{k_{i,h}^{t-1},k_{i,h}^{t-1}+\left(1-k_{i,h}^{t-1}\right)\;\tau \;sim\left(v_{d},s_{i}\right)\right\}$$

## 6. Experimental Results

To evaluate the performance of the proposed tracking system, we conducted an explicit benchmark using a dataset of persons guided by a robot, similar to the actual application in the hospital. This has been done in order to get comparable quantitative figures and to emphasize the necessity of a person re-identification in the given scenario and that the implemented face- and appearance-based re-identification in the proposed tracking framework works. In addition, logs of the sessions of our real-world user trials in the hospital have been analyzed to show that the system performs well in the intended application.

### 6.1. Benchmark on Labeled Dataset

**Figure 5:**
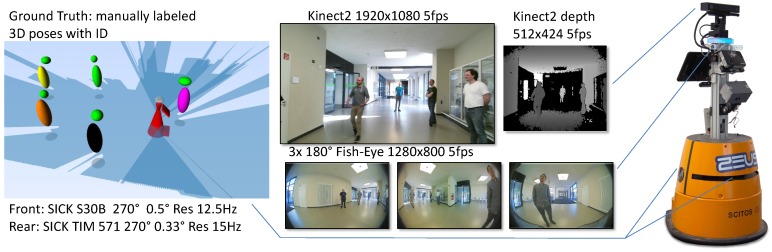
Dataset used for evaluation and the robot which has been used for recording (published in [22]) The sensor equipment for recording the dataset was similar that of the robot used in the real training application (see Figure 1) except the mounting point of the Kinect2 RGB-D camera.

For benchmarking, we used our multi-sensor dataset that has been published in [22]. The sensors used were the Kinect2, three fish-eye RGB cameras, and two SICK laser range finders as shown in Figure 5. The data comprise five guided tours in a public building similar to the hospital. In each of the trials one person followed the robot, while up to six additional persons walked around randomly. The dataset has been labeled manually, such that for each frame the ground truth position and orientation of the visible persons is known. For actual calculation of the online tracking results, the records have been replayed on the actual hardware of our target robot and all detections have been processed in real-time.

Following the method of our previous work [22], we use the Multiple Object Tracking Accuracy (MOTA) metric (see Equation (8)) from [35] to describe the completeness of the modeled state of persons in the robot’s surroundings. For every frame, that method uses the Hungarian Method to assign exactly one tracked hypothesis to each ground truth position using a maximum valid assignment distance of 1m. Ground truth positions which have no assigned hypothesis count as missed (*miss*). Likewise, hypotheses having no assigned ground truth count as false positives (*fp*). When a hypothesis is assigned to a ground truth with another ID than in the previous frame, an ID switch (*ids*) is counted. The values of these three measures are summed up over all *t* frames and divided by the number *g* of all ground truths positions to make sequences with a different number of persons comparable. The resulting MOTA values can range from -∞ (since the amount of false positives is theoretically unbound) to 1 for a perfect tracking of all ground truths.

(8) $$MOTA=1-\frac{\sum_{t}\left(miss_{t}+fp_{t}+ids_{t}\right)}{\sum_{t}g_{t}}$$

In addition to MOTA, we also calculate the Multiple Object Tracking Precision (MOTP) metric [35] which evaluates the spatial accuracy of our position tracker. The MOTP is calculated by the sum of total positional distances between matched hypothesis *i* and corresponding ground truth for every frame *t* averaged by sum *c* of matches found (see Equation (9)).

(9) $$MOTP=\frac{\sum_{i,t}d_{i,t}}{\sum_{t}c_{t}}$$

**Figure 6:**
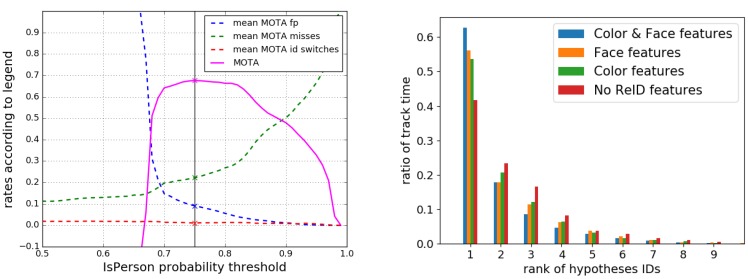
**Left**: Evolution of MOTA values with different existence thresholds. While higher thresholds lower the amount of false positives and ID switches, the amount of missed hypotheses increases. The threshold with the highest MOTA determines the best working point for our tracker in the application phase (vertical black line). **Right**: Sorted histograms for hypothesis IDs assigned to ground truth IDs. Colors indicate different combinations of features used. It can be seen, that by using more complex features, the values in the first bin almost doubles compared to a tracker without re-identification features. This first bin reflects the proportion of time a person has the correct ID, even if s/he has left the field of view in between. This is important for a robust re-identification of the target user in our gait training application.

Similar to [22], we tested the system by varying the threshold for the IsPerson probability (see Figure 6 left). This is necessary since our tracker also holds hypotheses of weak detections that have not been proved by subsequent detections of other sensors. Since the system models the confidence of the hypotheses in form of the IsPerson probability we can decide afterwards how optimistic our system should be by searching for the optimum threshold. If the probability of a hypothesis exceeds the given threshold, the hypothesis is considered as certain and used for the evaluation. If the probability is too low, it is not further processed. With the detector configuration presented in Section 3.1, we achieved a maximum MOTA score of 0.676 at a threshold of 0.75 for the certainty. This is comparable (0.11 points worse) to the best detector configuration from [22] which has been achieved using a large set of different detector modules in order to evaluate them. For the MOTP, we achieve an accuracy of 16.5cm on our dataset.

The proposed method of optimizing the threshold indicates that the system is relatively robust with respect to changes in parameters. The plateau in the MOTA curve shows that. For the other parameters manually selected values have been used in the first hand, while the similarity radius $r_{max}$ and parameters of the motion model have been optimized in a grid search without showing significant improvements.

In this paper, we extended the evaluation of MOTA and MOTP with a new metric for the person re-identification task, which evaluates the consistency of assigned IDs for each of the ground truth persons. Although, the ID switches are already counted in the MOTA, this measure cannot distinguish if the switch was from or to the correct ID. In order to find the contribution of both feature tracker modules, we compared the methods for re-identification using face and color appearance features one at a time and in combination, as well as no re-identification as baseline. A measure for the success of a re-identification after losing the track is the amount of time, a ground truth person has a consistent hypothesis ID. Which ID exactly does not matter since this is attached by the tracker. Therefore, for each ground truth person, we counted the associated hypothesis IDs in a histogram. Then, all bins were sorted in a descending order. Afterwards, the sorted histograms of all persons were added and then normalized to a sum of 1 in order to average over the complete dataset.

The amount of time a person is tracked with one consistent track ID is reflected in the first bin of the histogram. The other bins reflect the amount of time the tracker needs to identify a person when a track got lost and the person is detected again.

Over all guiding runs, without re-identification features in the tracker, the longest tracks cover only 42% of the time a person was present. This characterizes the dataset, in which persons walked in the office building randomly leaving the range of the cameras every now and then and reappearing behind doors and corners. Only one person was following the robot in a distance of about 3 m in the runs of the dataset. Since the evaluation, however, considered all persons and not only the guided one, the results are worse than for the real clinical scenario, where only the following patient needs to be tracked consistently.

Using the color appearance feature tracker, the time with a consistent hypothesis ID increases to 54%. Since the color features were extracted form the panorama cameras covering the full 360°, the low increment indicates a worse selectivity of the color compared to the face features, which could increase the track time to 56% while only operating on the Kinect2 images with the small view angle. Persons walking in front of the robot or by side cannot be identified by means of their faces. The value of 63% track time for the combined case using both feature trackers shows that both systems complement each other synergetically.

A detailed analysis of the systems accuracy with respect to persons’ orientation on the same dataset can be found in [32]. That study shows that a tracking brings a real benefit over just taking the frame wise detection results. Also for results of the posture estimation the interested reader is referred to a separate study [24].

### 6.2. Real-World User Trials

Besides the theoretical evaluation on a test dataset, the performance of the system in real world has been evaluated during user trials of the complete application interacting completely autonomous. During user tests in our target clinic scenario both observations, face and color appearance features, have been used in the tracker. These tests took place between August and September 2019 at the Waldklinik Eisenberg in Thuringia, Germany. 22 patients repeatedly were guided by the robot along a corridor of the clinic (see Figure 1). At each point, the patients were able to terminate the exercise by sitting down, engaging a dialogue and walk back to the beginning of the training course. At 16 training days, the 22 patients performed 95 walking exercises with an overall training duration of 11.6 h. During the whole time, the robot drove 17.8 km and encountered 458 persons (excluding the users). During all exercises, the user was lost for longer than 10 s only 11 times. In these cases, the robot went back to the last known place of its patient and waited until the training was restarted by a new login at the GUI. The tracking results together with the user-adaptive navigation and camera control could achieve that 86% of the training time the patient has been kept in view of the Kinect2 camera for analysis of the gait patterns. Details on the navigation and camera controller can be found in [36].

## 7. Conclusions

We presented a tracking framework that solves several problems arising from a multi-sensor setup on a mobile platform; namely, asynchronous delayed observation from diverse detection methods having individual false detections and miss rates. The application in a public hospital environment requires a re-identification of the current user after interruptions of the track have occurred. This was realized implicitly in the tracker by means of the modular concept allowing us to track appearance-based features and facial features in addition to the actual position and other time variant properties also necessary for a socially acceptable robot navigation. The proposed fusion of data-association decisions of the individual modules leads to the re-identification of persons seen in the past. The experiments using a labeled multi-sensor dataset showed that the consistency of track IDs improved significantly when face and appearance features were included in the tracking system. This improvement enabled us to realize our rehabilitation assistant which successfully completed a three week user trial.
